# Influence of Intramuscular Injection Sites on Pharmacokinetics of Amoxicillin in Olive Flounder (*Paralichthys olivaceus*) and Its Implication for Antibacterial Efficacy

**DOI:** 10.3390/pharmaceutics15041153

**Published:** 2023-04-05

**Authors:** Ji-Hoon Lee, Ga-Won Kim, Hyun-Woo Kang, Joo-Won Hong, Hyo-Eun Lee, Mun-Gyeong Kwon, Jung-Soo Seo

**Affiliations:** Aquatic Disease Control Division, National Fisheries Products Quality Management Service, 216 Gijanghaean-ro, Gijang-eup, Gijang-gun, Busan 46083, Republic of Korea

**Keywords:** amoxicillin, injection site, olive flounder, pharmacokinetics, PK/PD

## Abstract

This study aimed to investigate the effects of different injection sites, including dorsal, cheek, and pectoral fin muscles, on the pharmacological properties of amoxicillin (AMOX) in olive flounder (*Paralichthys olivaceus*) after a single intramuscular (IM) injection of 40 mg/kg. The AMOX concentration was measured using high-performance liquid chromatography-tandem mass spectrometry, followed by a non-compartmental model analysis. The peak serum concentrations (C_max_) achieved 3 h after dorsal, cheek, and pectoral fin IM injections were 202.79, 203.96, and 229.59 μg/mL, respectively. The area under the concentration-time curve (AUC) was 1697.23, 2006.71, and 1846.61 µg/mL·h, respectively. The terminal half-life (t_1/2_λ_Z_) was prolonged for cheek and pectoral fin IM injections (10.12 and 10.33 h, respectively) compared to dorsal IM injection (8.89 h). In the pharmacokinetic-pharmacodynamic analysis, a higher T > minimum inhibitory concentration (MIC) and AUC/MIC values were observed after AMOX was injected into the cheek and pectoral fin muscles compared to the dorsal muscle. Muscle residue depletion was below the maximum residue level from day 7 after IM injection at all three sites. These findings suggest that the cheek and pectoral fin sites provide advantages regarding systemic drug exposure and prolonged action compared with the dorsal site.

## 1. Introduction

In the aquaculture industry, antibiotics are commonly administered via injection for the olive flounder (*Paralichthys olivaceus*) owing to the advantages it offers over oral administration in certain circumstances [1,2]. Compared to other farmed fish species, the olive flounder has a calm demeanor and flat body, which makes physical injection of drugs feasible [1]. In Korea, injectable formulations of amoxicillin, lincomycin, and tylosin have been developed for aquaculture use [3] (https://www.nfqs.go.kr/apms/ebook/mice_ebook/index.html#page=1, accessed on 25 February 2023). The approved injection site for these antibiotics is only the dorsal muscle, which is the main edible part, thus necessitating the exploration of other sites for more effective disease treatment and food safety.

Amoxicillin (AMOX) is a member of the semisynthetic penicillin (β-lactam class) group, which mainly acts on gram-positive bacteria by inhibiting bacterial-cell-wall synthesis, leading to death due to osmotic rupture. In Korea, AMOX is approved for a single intramuscular (IM) dose of 40 mg/kg to treat streptococci caused by *Streptococcus iniae* and *S. parauberis* in olive flounder. Limited studies have been conducted to determine the pharmacological characteristics of sodium and trihydrate salts of AMOX in olive flounder. Lim et al. [4] and Park et al. [5] reported a pharmacokinetic-pharmacodynamic (PK-PD) relationship by integrating PK and minimum inhibitory concentration (MIC) data after a single IM injection of AMOX sodium salt into olive flounder. Additionally, regarding food safety, a withdrawal time has been suggested using the muscle residue depletion test [4,5]. Seo et al. [6] determined the PK of AMOX in olive flounder after a single IM injection of trihydrate salt.

The extent of drug absorption after IM injection depends not only on physiochemical characteristics, such as lipid solubility and pKa, but also on factors such as the vascularity of the injection site [7]. Therefore, administering drugs to various injection muscle sites may cause concentration differences in the body, depending on the branch of the central blood vessel as well as blood flow distribution owing to the type of muscle. This study aimed to investigate the influence of three different injection sites on the PK profile and PK-PD relationships of AMOX after a single IM injection in olive flounder.

## 2. Materials and Methods

### 2.1. Drug and Chemicals

A parenteral formulation of AMOX injection (amoxicillin sodium, water-soluble powder, 50 g) was obtained from KBNP Veterinary Medicine Co., Ltd. (Yesan, Chungnam-do, Korea) and diluted with phosphate-buffered physiological saline to achieve a final injection concentration of 40 mg/kg. The mobile phase for high-performance liquid chromatography-tandem mass spectrometry (HPLC-MS/MS) was purchased from Merck (Whitehouse Station, NJ, USA). The following chemicals were purchased from Sigma (St. Louis, MO, USA): amoxicillin, sodium, piperacillin, acetic acid, sodium phosphate dibasic (Na_2_HPO_4_), potassium dihydrogen phosphate (KH_2_PO_4_), and formic acid. For solid-phase extraction (SPE), an OASIS^®^ PRiME HLB 6 cc (200 mg) Extraction Cartridge was purchased from Waters (Milford, MA, USA).

### 2.2. Experimental Animals

Healthy olive flounder, *Paralichthys olivaceus*, weighing 408.2 ± 35.7 g (body length, 33.1 ± 1.5 cm) were purchased from a private farm in Tongyeong city, Gyeongnam, Korea, and acclimated for 3 weeks under laboratory conditions. The fish were fed a commercial pellet diet (CJ Feed Inc., Gunsan, Jeonbuk, Korea) at a daily rate of 1.5% in running water-type culture tanks (120 L). The natural seawater was sterilized with UV before introduction, and the temperature was maintained at 22 ± 0.5 °C using heat-controlling systems (Aquatron System, Yuone Electric, Korea). The water quality was assessed daily at 10:00 AM using a portable analyzer (YSI, Yellow Springs, OH, USA) to maintain approximately 30 ppt salinity, 8 mg/L dissolved oxygen, and a pH of 7.8. Before drug administration, the fish were sacrificed to confirm the absence of amoxicillin in the serum, muscle, and skin. No fish died during the experiments. All fish experiments were approved by the Institutional Animal Care and Use Committee (IACUC, NFQS-2022-6).

### 2.3. Experimental Design

**Figure 1:**
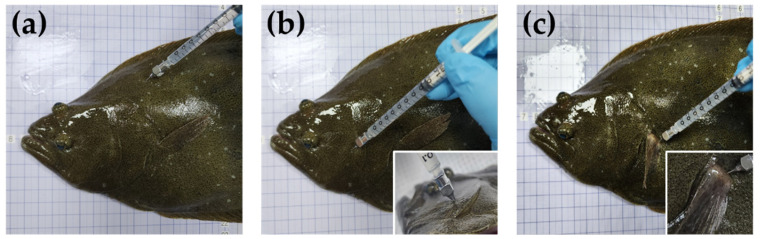
Representative photo describing the intramuscular injection sites of amoxicillin in olive flounder (*Paralichthys olivaceus*): (**a**) dorsal, (**b**) cheek, and (**c**) pectoral fin muscles.

Olive flounders were randomly divided into three equal groups of 70 individuals each. In a parallel study, fish in the first group were administered a single IM dose of 40 mg/kg (currently approved in Korea) into the dorsal muscles. Olive flounders from the second and third groups were injected with the same dose into the cheek and pectoral fin muscles, respectively. The three different IM injection sites are shown in Figure 1. For the PK analysis, blood samples (approximately 1.2 mL) were collected from the caudal vein at 0.5, 1, 3, 6, 12, 24, 48, and 72 h after drug administration and transferred to a serum separator tube. The blood samples were centrifuged at 3000× *g* for 10 min at 4 °C. For residue depletion analysis, muscle and skin samples were obtained 1, 2, 3, 7, and 20 d after drug administration. All samples were stored at −80 °C until analysis. Drug administration and blood sampling were conducted without the use of anesthetics to avoid potential drug–drug interactions [8,9].

### 2.4. HPLC-MS/MS and Sample Preparation

Serum and muscle concentrations of AMOX were determined using an Agilent 1260 Infinity series liquid chromatograph coupled with an Agilent 6420 Triple Quad detector (Agilent Technologies, Santa Clara, CA, USA). Chromatographic separation was performed using a Waters XSelect HSS C₁₈ (2.1 mm × 150 mm, 3.5 μm) maintained at 35 °C. The mobile phase consisted of A (0.05% formic acid in water) and B (0.05% formic acid in ACN) with gradient elution as follows: 0–10 min, linear gradient to 50% B; 10–15 min, linear gradient to 75% B; 15–15.1 min, linear gradient to 10% B, 15.1–20 min, hold at 10% B at a flow rate of 0.2 mL/min. The mass spectrometer was run in multiple reaction monitoring modes for positive charges focused on AMOX, both as a quantifier (*m*/*z* 366.2–348.9) and qualifier (*m*/*z* 366.2–114).

The samples were treated according to the method developed by the Korean Food Standards Codex [10] (https://residue.foodsafetykorea.go.kr/vd/analysis, accessed on 26 February 2023) for fish food. Homogenized samples (1 mL of serum or 2 g of muscle + skin) were transferred to 50 mL conical polypropylene tubes, and after adding 1 mL of 0.1 N acetic acid, the samples were immediately sonicated (60 Hz, 300 W) for 5 min at 25 °C. Next, 8 mL of methanol and 200 µL of internal standard (piperacillin, 500 ppb) were added to the samples, followed by centrifugation at 20,000× *g* at 4 °C for 10 min. The clear supernatant was transferred to 15 mL conical polypropylene tubes, and the extract was evaporated using a stream of nitrogen at 50 °C. Then, 5 mL of 0.1 M Na_2_HPO_4_ (pH 7.2) was added to the evaporation residue. The mixture was loaded onto 0.05 M Na_2_HPO_4_ pre-conditioned (5 mL pre-rinsing) Hydrophilic-Lipophilic Balance (HLB) cartridge columns, rinsed with 5 mL of 0.05 M Na_2_HPO_4_, and eluted with 5 mL of 0.05 M KH_2_PO_4_:acetonitrile (50:50, *v/v*). The eluted extract was evaporated using a stream of nitrogen at 50 °C. The residue was reconstituted with 1 mL of water and filtered using 0.2-µm polyvinylidene fluoride (PVDF) syringe filters. Twenty microliters of the residue were added to the HPLC-MS/MS system.

This assay was thoroughly validated using limits of quantitation of 25 and 10 ng/mL (g) in serum and muscle, respectively. Matrix-matched calibration (MMC) showed a strong linear regression, and an MMC range of 10–500 ng/mL (g) was attained for this analysis. The inter-day coefficient of variation was below 12.12% for the three concentrations (1 LOQ, 5 LOQ, and 10 LOQ). The average recovery rates ranged from 95.44% to 109.92%, satisfying the criteria of the Bioanalytical Method Validation Guidance for Industry [11].

### 2.5. Pharmacokinetic Analysis

A non-compartmental model based on statistical moments theory was used to perform a PK parameter analysis of the serum AMOX concentration. The analysis was conducted using the PKSolver software add-in for Microsoft Excel [12]. The peak serum concentration (C_max_) and time to C_max_ (T_max_) were determined directly from the experimental data. The terminal rate constant (λ_z_) was calculated using a linear regression of the logarithmic serum concentration, and the terminal half-life (t_1/2_λ_z_) was calculated using the equation ln2/λ_z_. The area under the concentration-time curve (AUC) and the area under the first moment curve (AUMC) were calculated using the linear trapezoidal method. Finally, the mean residence time (MRT) was calculated as AUMC divided by the AUC.

### 2.6. Pharmacokinetic/Pharmacodynamic Relationships

MICs that inhibit 90% of growth (MIC_90_) for olive flounder have been reported. MIC_90_ values of 0.0312 and 0.5 µg/mL were previously described for *Streptococcus iniae* and *S. parauberis* isolated from olive flounder, respectively [4]. These values were used to calculate the PK/PD parameters. Surrogate markers for antibacterial efficacy were determined by calculating the ratios of C_max_/MIC_90_, AUC/MIC_90_, and T > MIC_90_ [1,13] following the three intramuscular injection sites.

### 2.7. Statistical Analysis

Statistical analyses of the PK parameters between different groups were not performed except for C_max_ because the fish had a relatively low body weight, and blood samples were not continuously collected from individual fish. Therefore, only one final value was obtained for each PK parameter, which was calculated based on the average concentration versus time data. Given that C_max_ was derived from seven fish after IM, the standard deviation was obtained, and statistical analysis was performed using GraphPad Prism version 5.03 software (GraphPad Software, Inc., La Jolla, CA, USA). For nonparametric data (C_max_), significant differences between three groups were determined using the Kruskal–Wallis test, followed by Dunn’s multiple comparison test; *p*-values < 0.05 were considered statistically significant.

## 3. Results

### 3.1. Serum Pharmacokinetics

**Figure 2:**
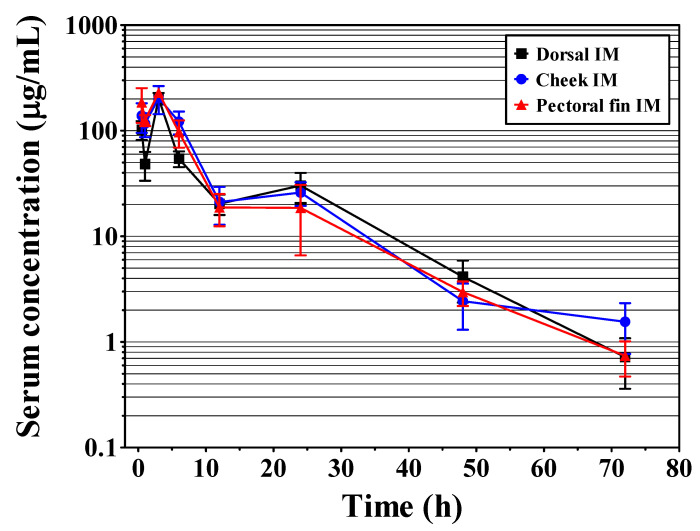
Semilogarithmic of serum concentration-time curves of amoxicillin in olive flounder (*Paralichthys olivaceus*) following intramuscular (dorsal, cheek, and pectoral fin) injection at a dosage of 40 mg/kg.

No behavioral abnormalities or signs of toxicity were observed in olive flounder after IM AMOX injection at any site (dorsal, cheek, and pectoral fin muscles). The serum concentration-time curves of AMOX after IM injection and the calculated PK parameters are presented in Figure 2 and Table 1, respectively. At dorsal, cheek, and pectoral fin IM injections, the C_max_ of AMOX were 202.79, 203.96, and 229.59 μg/mL, respectively, and the corresponding T_max_ was 3 h in all cases. The AUC and t_1/2_λ_Z_ were greater in the cheek and pectoral fin muscles than in the dorsal muscle. Specifically, at dorsal, cheek, and pectoral fin muscles, the AUCs of AMOX were 1697.23, 2006.71, and 1846.61 µg/mL·h, respectively, and the t_1/2_λ_Z_ were 8.89, 10.12, and 10.33 h. The other parameters are summarized in Table 1.

### 3.2. Pharmacokinetic/Pharmacodynamic Relationships

The ratios of C_max_/MIC_90_, AUC/MIC_90_, and T > MIC_90_ were determined by integrating the serum PK parameters with the MIC_90_. The largest AUC/MIC_90_ and T > MIC_90_ ratios were determined for *Streptococcus iniae* and *S. parauberis* for cheek IM injections, while the C_max_/MIC_90_ ratios were the greatest for pectoral fin IM injections (Table 2).

### 3.3. Muscle Residue Depletion

AMOX concentrations in olive flounder muscle and skin following AMOX IM injection are shown in Table 3. Rapid declines in residue concentrations were detected in olive flounder following AMOX IM injection at all sites (dorsal, cheek, and pectoral fin), with levels ranging from 1.28 to 0.06 mg/kg, 2.20 to 0.11 mg/kg, and 1.04 to 0.08 mg/kg, respectively, from day 1 to day 3 post-injection. At day 7 post-injection, no samples exceeded the MRL of 0.05 mg/kg of AMOX.

## 4. Discussion

To the best of our knowledge, this study is the first to investigate the effect of IM injection sites on the pharmacological profile of AMOX in fish, specifically in olive flounder. However, the effects of various IM injection sites on PK have been previously reported in terrestrial animals, such as piglets, sheep, horses, and geese [15,16,17,18]. Sooud et al. [18] demonstrated that IM injection of marbofloxacin into the thigh muscle of geese produced a higher maximum drug concentration than injection into the pectoral muscle. Furthermore, when penicillin G was injected into horses, the neck area was found to be advantageous for drug absorption [17], and a similar trend was observed when AMOX was injected into sheep [16]. Based on the findings from previous studies on terrestrial animals, this study aimed to explore the potential pharmacological advantages of using alternative injection sites in olive flounder, as opposed to the dorsal muscle, which is the main injection site.

The rate and extent of drug absorption may differ owing to vascularity at the injection site and the characteristics of the inter- or intramuscular blood supply [16]. Injection of AMOX into the cheek and pectoral fin muscles via the IM route resulted in similar or higher C_max_ values compared to the dorsal muscle (dorsal muscle, 202.79 μg/mL; cheek muscle, 203.96 μg/mL; pectoral fin muscle, 229.59 μg/mL). The t_1/2_λ_Z_ was longer with IM administration to the cheek (10.12 h) and pectoral fin (10.33 h) muscles than to the dorsal muscle (8.89 h). Owing to the high C_max_ and slow elimination, administration of AMOX to the cheek and pectoral fin muscles resulted in a significantly greater systemic exposure (AUC_0-t_ = 2006.71 and 1846.61 μg/mL·h, respectively) compared to dorsal injection (AUC_0-t_ = 1697.23 μg/mL·h). The differences in these PK parameters may be attributed to differences in vascularity and blood flow in the cheek and pectoral fin muscles compared to those in the dorsal muscles, as previously mentioned. Most dorsal muscles in olive flounder are white muscles [19], which have smaller blood volumes and blood vessels than red muscles [20]. In contrast, the cheek muscles in fish have a better blood flow than regular white muscles [21], and the pectoral fin along the pectoral girdle is well-vascularized by the arterial subclavian branches and vena brachialis [22]. These findings explain the differences in the PK characteristics of the three different injection sites.

T > MIC is a crucial predictor of the effectiveness of time-dependent antibacterial agents such as AMOX and can serve as a PK/PD surrogate [23]. The MIC_90_ values of AMOX were 0.0312 and 0.5 μg/mL for the two most susceptible microorganisms (*S. iniae* and *S. parauberis*, respectively) that cause severe disease in farmed olive flounder in Korea [4]. This study showed that a longer T > MIC was observed after AMOX injection into the cheek and pectoral fin muscles than after injection into the dorsal muscle, indicating that the cheek and pectoral fin muscles are suitable injection sites for prolonged drug activity. It has been established that AUC/MIC ratios ≥ 25–30 and C_max_/MIC ratios ≥ 10–12 are major determinants of the clinical efficacy of antibacterial drugs to obtain generally satisfactory clinical treatment results [24]. Overall, greater AUC/MIC and C_max_/MIC ratios were achieved in the cheek and pectoral fin muscles than in the dorsal muscles, although all three injection sites showed significant clinical efficacy.

Residue depletion analyses demonstrated a rapid depletion of AMOX in the edible tissues of olive flounder. The Ministry of Food and Drug Safety [14] has established the MRL of AMOX in fish that is safe for human consumption to be 0.05 mg/kg. The detection of AMOX was below the MRL on day 7 after dosing at all three injection sites. This is advantageous for the treatment of edible fish using the cheek and pectoral fin muscles, which were first used as injection sites for AMOX in olive flounder.

This study had some limitations. First, blood samples were not consistently collected from individual fish; a statistical analysis of PK parameters between different groups would optimize drug use recommendations. Second, these results are based on the PK data obtained following AMOX injection into healthy olive flounder. Although injectable antibiotics can be used in aquaculture farms for diseased fish, the appropriate dosages for diseased fish cannot be predicted. Therefore, further studies on healthy and diseased olive flounder using continuous blood sampling from individual fish are required to optimize injection site recommendations for AMOX use and improve clinical outcomes associated with the use of this drug in olive flounder.

## 5. Conclusions

Overall, the PKs of AMOX were significantly influenced by the cheek and pectoral fin muscle injection sites, compared to the dorsal muscles, in olive flounder. Moreover, PK/PD relationships were established for each injection site against major clinical strains, and food safety was confirmed through residue depletion analysis. To the best of our knowledge, this study is the first to investigate the impact of injection sites on the PK profile of AMOX in fish, specifically in olive flounder. As a new injection site, the cheek and pectoral fin muscles of the olive flounder may provide a more effective and safer clinical treatment method than the dorsal muscle.

## Figures and Tables

**Table 1:** Pharmacokinetic parameters of amoxicillin in serum of olive flounder (*Paralichthys olivaceus*) following intramuscular (dorsal, cheek, and pectoral fin) injection at a dosage of 40 mg/kg.

PK Parameters	Unit	Dorsal IM	Cheek IM	Pectoral Fin IM
λ_z_	h^−1^	0.08	0.07	0.07
t_1/2_λ_z_	h	8.89	10.12	10.33
T_max_	h	3.00	3.00	3.00
C_max_ *	µg/mL	202.79 ± 24.88	203.96 ± 58.26	229.59 ± 26.70
AUC_0–24_	µg/mL·h	1227.19	1618.71	1540.42
AUC_0-t_	µg/mL·h	1697.23	2006.71	1846.61
AUC_0-inf_	µg/mL·h	1706.42	2029.39	1854.69
AUMC_0-inf_	µg/mL·h^2^	24,469.78	24,582.22	19,622.66
MRT_0-inf_	h	14.34	12.11	10.58

* The peak serum concentration (C_max_) after intramuscular injection was derived from seven fish; thus, the standard deviation could be obtained. Data are expressed as the mean of the seven olive flounder species. λ_z_, first-order rate constant associated with the terminal portion of the curve; t_1/2_λ_z_, terminal half-life; T_max_, time to peak serum concentration; C_max_, peak serum concentration; AUC, area under the concentration-time curve; AUMC, area under the first moment curve; MRT, mean residence time; IM, intramuscular.

**Table 2:** Pharmacokinetic/pharmacodynamic integration of amoxicillin in serum of olive flounder (*Paralichthys olivaceus*) following intramuscular (dorsal, cheek, and pectoral fin) injection at a dosage of 40 mg/kg.

PK/PD Parameters	Dorsal IM	Cheek IM	Pectoral Fin IM
*Streptococcus iniae*			
C_max_/MIC_90_	6499.68	6537.18	7358.65
AUC_0–24_/MIC_90_ (h)	39,333.01	51,881.73	49,372.44
AUC_0-t_/MIC_90_ (h)	54,398.40	64,317.63	59,186.22
AUC_0-inf_/MIC_90_ (h)	54,692.95	65,044.55	59,445.19
T > MIC_90_ (h)	111.61	132.37	118.28
*Streptococcus parauberis*			
C_max_/MIC_90_	405.58	407.92	459.18
AUC_0–24_/MIC_90_ (h)	2454.38	3237.42	3080.84
AUC_0-t_/MIC_90_ (h)	3394.46	4013.42	3693.22
AUC_0-inf_/MIC_90_ (h)	3412.84	4058.78	3709.38
T > MIC_90_ (h)	76.05	85.35	76.87

PK/PD parameter has been calculated for three intramuscular injection sites based on reported MIC_90_ (0.0312 and 0.5 μg/mL) for *Streptococcus iniae* and *S. parauberis* [4].

**Table 3:** Residue depletion of amoxicillin in muscle and skin of olive flounder (*Paralichthys olivaceus*) following intramuscular (dorsal, cheek, and pectoral fin) injection at a dosage of 40 mg/kg.

Time (day)	Amoxicillin Concentration (mg/kg)		
	Dorsal IM	Cheek IM	Pectoral Fin IM
1	1.28 ± 0.14 (7/7)	2.20 ± 1.17 (7/7)	1.04 ± 0.45 (7/7)
2	0.27 ± 0.12 (7/7)	0.24 ± 0.09 (7/7)	0.18 ± 0.06 (7/7)
3	0.06 ± 0.03 (5/7)	0.11 ± 0.04 (6/7)	0.08 ± 0.03 (6/7)
7	Not detected	Not detected	0.01 ± 0.01 (0/7)
20	Not detected	0.01 ± 0.02 (0/7)	Not detected

Data are expressed as mean ± SD values from seven olive flounders. The numbers in the parentheses denote the individual fish with amoxicillin concentrations above the MRL of 0.05 mg/kg [14].

## Data Availability

Not applicable.
